# The therapeutic potential of Camel Wharton jelly mesenchymal stem cells (CWJ-MSCs) in canine chronic kidney disease model

**DOI:** 10.1186/s13287-022-03076-8

**Published:** 2022-07-30

**Authors:** Hala M. F. El Miniawy, Haithem A. Farghali, Marwa S. Khattab, Ibrahim A. Emam, Essam M. Ibrahem, Dina Sabry, Tahany A. Ismail

**Affiliations:** 1 Department of Pathology, Faculty of Veterinary Medicine, Cairo University, ElGamaa street, Giza, 12211 Egypt; 2 Department of Surgery, Anesthesiology and Radiology Department, Faculty of Veterinary Medicine, Cairo University, Giza, Egypt; 3 Department of Medical Biochemistry and Molecular Biology, Faculty of Medicine, Cairo University, Giza, Egypt; 4 Animal Health Research Institute, Nadi El-Seid Street, Dokki, PO Box 264, Cairo, Giza, 12618 Egypt

**Keywords:** Chronic kidney disease, Camel stem cells, Histopathology, Kim-1, NGAL

## Abstract

**Background:**

Chronic kidney disease (CKD) is a worldwide health problem that its incidence increases nowadays with the increase in the risk of environmental pollution. CKD can progress to end-stage renal disease (ESRD) which usually ends fatally. This study aimed to examine the therapeutic potential of Camel Wharton jelly-mesenchymal stem cells (CWJ-MSCs) in chronic kidney disease model induced in dogs.

**Methods:**

CWJ-MSCs were injected directed to the kidney with ultrasonographic guidance in dogs with 5/6 nephrectomy to evaluate its therapeutic potency in such cases. Analysis of variance was applied in normally distributed quantitative variables while a non-parametric Mann–Whitney test was used for non-normally distributed quantitative variables.

**Results:**

The serum urea and creatinine in the treated group were significantly decreased transferring dogs in the treated group from stage 3 to stage 2 CKD according to the IRIS staging system. Histopathology of renal tissue revealed improving CKD lesions by increasing regeneration of degenerated tubules, maintaining the integrity of glomeruli. New vascularization with blood vessels remodeling were common findings. Periodic acid Schiff stain of renal tissue showed the integrity of renal tubules and thickness of the glomerular basement membrane. Fibrosis of cortex and medulla was lower in the treated group than in the CKD model as monitored by Mallory’s trichrome stain (MTC). NGAL and KIM-1 genes expression were decreased while VEGF and EGF genes expression were increased indicating renal tissue repair.

**Conclusions:**

CWJ-MSCs have a therapeutic potential in the CKD model induced in dogs.

## Background

Chronic kidney disease (CKD) is an irreversible process with progressive loss of renal function [1]. The kidney can regenerate after limited injury [2]. This regenerative potential is limited under chronic conditions and inefficient to prevent the progression of glomerulosclerosis and tubulointerstitial fibrosis [3]. Treatment strategies with cellular regeneration offer a good alternative therapy for patients with CKD. Several types of stem cells have been used in the treatment of kidney injury ranging from mesenchymal stem cells (MSCs) [4, 5], adipose stem cells (ADSCs) [6], amniotic fluid stem cells (AFSCs) [7, 8], and renal progenitors (RPs) [9, 10]. Compared to other stem cell types, MSCs were more efficient in slackening CKD development and progression [11]. The renoprotective activity of stem cells is due to stem cell secretion of cytokines that impede inflammation and fibrosis as well as trigger endogenous repair processes like angiogenesis. Recently, several studies demonstrated that stem cells may mediate their mode of action through extracellular vesicles (EVs) secretion [12, 13]. These vesicles can transfer genetic data to host renal cells initiating regenerative processes.

In preclinical studies, the 5/6 nephrectomy was considered a good model for chronic kidney diseases. It was applied successfully in rats [14, 15] and recently in dogs [16]. Few preclinical studies address the use of stem cells in large animal kidney injuries. For instance, the injection of autologous mesenchymal cells in sheep with ischemia–reperfusion injury did not repair kidney lesions as reported in similar rodent studies. Similarly, in the rhesus monkey, I/R injection of autologous MSCs in the preventive CKD monkey model delayed the progression of CKD but didn’t reduce established interstitial fibrosis in the stable CKD model [17].

Wharton’s jelly mesenchymal stem cells (WJ-MSCs) are more primitive and efficient MSCs than those derived from adult fat or bone marrow [18, 19]. Nevertheless, it is the mesenchymal stem cell type least used in experimental studies. There are very few studies that covered camel stem cells and their therapeutic effect [20], although camel stem cells have a promising role in regenerative medicine. El Miniawy et al. [21], succeeded to isolate the endothelial progenitor stem cells from camel peripheral blood and in vitro differentiation to osteoblast, chondrocyte, and neural cells. Moreover, CWJ-MSCs succeeded to alleviate the induced diabetes by a significant increase in quantitative genes expression, enhanced the histopathology of islets of Langerhans, pancreatic acini, and improved serum urea level and histopathology of kidney in a diabetic rat model [22]. This study aimed to investigate the therapeutic potential of CWJ- MSCs in chronic kidney disease model induced in dogs as an example for the usage of large animals in preclinical studies, as large animal models remain important for translation.

## Materials and methods

### Animals

A total number of (6) male Mongrel dogs (age: 2–3 years; weight: 20–25 kg) were purchased from a local breeder. They were housed in kennels of Surgery, Anesthesiology and Radiology Department, Faculty of Veterinary Medicine, Cairo University, and maintained at a constant temperature. Animals had free access to a standard diet and drinking water. The experimental animals were divided into 2 equal groups.

### Camel Wharton’s Jelly mesenchymal stem cells (CWJ-MSC) preparation

Umbilical cord specimens were obtained fresh from a healthy pregnant camel and placed in sterile saline at 4 °C. The cord was washed several times with sterile saline and cut into 2–4 cm lengths. The cord was opened after stripping the vessels manually and the tissue was digested for 60 min in (3 mg/ml) collagenase II enzyme (Sigma; USA) at 37 °C. Tissue debris was removed from strained digested tissue and then centrifuged at 250 × g for 5 min at room temperature. A complete culture medium formed of Dulbecco's modified Eagle’s medium (DMEM), 10% fetal bovine serum (FBS), and antibiotics (100 U/mL penicillin and 100 μg/mL streptomycin) was used for suspension of strained cells. Cells were incubated for 12–14 days at 37 °C in 5% humidified CO2 and were considered as passage 0 [23]. Isolated CWJ-MSC was identified by morphology and flow cytometric analysis. Trypsin was used to harvest cultured MSCs which were then stained with specific antibodies (CD 90 and CD105) following BD- Biosciences, USA protocol and analyzed by Flow Antibody Cell Sorting (FACS) Calibur flow cytometer [24].

### Induction of CKD surgically by 5/6 nephrectomy

Each experimental group was subjected to laboratory screening including a complete blood cell count (CBC), urinalysis (UA), and serum biochemistry, to evaluate the general health conditions and normal kidney function of the animals under the experiment and to detect subclinical disease such as hypoproteinemia and anemia that may affect the animal response to anesthesia [25]. Complete aseptic preoperative preparations of the operation site were done according to Bigliardi et al. [26].

G (1): CKD was induced in three dogs by excision of two poles (5/6 nephrectomy) through surgical removal of the upper and lower pole of the right kidney and contralateral nephrectomy after two weeks from the first surgical operation according to Ismail et al. [16].

G (2): CKD was also induced in the three dogs as in G1 and injected twice with undifferentiated CWJ-MSC at a dose (5 × 10^6^ cells) directly in the renal cortex guided by ultrasonography. The time interval between the two injections was two weeks [27].

### CWJ- MSCs transplantation by ultrasound-guided renal injection

Dogs were anesthetized and ultrasonography examination was done in dorsal recumbency using multifrequency 7.5 MHz real-time curved transducer (Sonoace R3, Korea). Ultrasound-guided renal injection of CWJ- MSCs were directly injected into one site of the renal cortex at 3rd week after the first surgery and the second dose after two weeks from the first dose [27]. CWJ MSCs transplantation was performed at the diagnostic image unite, Department of Surgery, Anesthesiology, and Radiology, Faculty of Veterinary Medicine, Cairo University, Egypt.

### Evaluation of renal function

Before the first operation, blood samples were taken from each group to estimate the serum urea and creatinine level, it was considered the zero time. Every week blood samples were collected from the jugular vein till receiving the second dose of CWJ MSCs then the blood samples were taken every two weeks. Serum Urea and creatinine were measured using commercial kits (Spectrum, Egypt) according to manufacture protocol.

### Histopathology of the kidney

After 9 weeks from the first stem cells injection, dogs were humanely euthanized by intravenous injection of a fully saturated solution of potassium chloride, and 20% solution of pentobarbital sodium [28]. Tissue specimens of the kidneys were collected from euthanized dogs and excised kidney poles from the first operation (normal control). Tissues were kept in 10% neutral buffered formalin for fixation, processed by ethanol and xylene, and embedded in paraffin. Tissue Sects. (3–4 µm) were stained with Hematoxylin and Eosin [29], Periodic acid Schiff reaction (PAS) was counterstained with Meyers’s hematoxylin [30], and Mallory’s trichrome stain (MTC) [31]. Image analysis of the area percent of positive PAS and MTC stained tissue was performed on 5 images 200 × magnification power /dog using image J software.

### Histopathological scoring of the kidneys

Semiquantitative scoring of the severity of glomerular sclerosis (GS) and tubular injury (TI) was performed based on a previous scoring system [32]. Briefly, the GS score was evaluated as follows: 0, normal GS; 1, matrix expansion of glomeruli or GS < 25%; 2, GS = 26–50%; 3, GS = 51–75%; and 4, GS > 75%. The TI score is as follows: 0, normal; 1, mild fibrosis around the vasculature; 2, mild fibrosis around the tubules; 3, moderate fibrosis with tubular casts or tubular damage; and 4, severe fibrosis with cells infiltration.

### Immunohistochemistry

Immunohistochemistry of vascular endothelial growth factor (VEGF) was performed on paraffin-embedded renal tissue using a primary antibody against VEGF (anti-VEGF, Lab Vision, USA), anti-species secondary antibody (biotinylated goat anti-rabbit), and an avidin–biotin-peroxidase complex kit according to manufacturer protocol (Histostain SP kit, Zymed Laboratories Inc, San Francisco, USA) [33]. Image analysis of VEGF expression was performed on 5 images 200 × magnification power/dog using image J software.

### Real-time PCR for Kim-1, NGAL & EGFR genes

Total RNA was extracted from homogenized tissues with Direct-zol RNA Miniprep Plus (Cat# R2072, ZYMO RESEARCH CORP. USA) and then quantified by Beckman dual spectrophotometer (USA).

Reverse transcription of extracted RNA was performed followed by PCR using SuperScript IV One-Step realtime-PCR (RT-PCR) kit (Cat# 12594100, Thermo Fisher Scientific, Waltham, MA USA). The thermal profile employed in 48-well plate StepOne instrument (Applied Biosystem, USA) was as follows: 10 min at 45 ºC for RT, 2 min at 98 ºC for RT inactivation and initial denaturation by 40 cycles of 10 s at 98 ºC, 10 s at 55 ºC and 30 s at 72 ºC for the amplification step. The data were expressed in Cycle threshold (Ct) for the target genes and housekeeping gene after the RT-PCR run. Normalization for variation in the expression of each target gene; Neutrophil gelatinase-associated lipocalin (NGAL), kidney injury molecule (KIM-1), and Epidermal growth factor receptor (EGFR) was carried out related to the mean critical threshold (CT) expression values of the GAPDH housekeeping gene by the ΔΔCt method. The relative quantitation (RQ) of each target gene is quantified following the calculation of the 2-∆∆Ct method. Primers sequence for NGAL gene was; forward 5′-GGTACGTCATCGGCATAGCA-3′ and reverse 5′-CCTGAGTAGGGTGGAGGTGA-3′, for KIM-1 gene, was; forward 5′‐ATGAATCAGATTCAAGTCTTC‐3′ and reverse 5′‐TCTGGTTTGTGAGTCCATGTG‐3′ for EGFR gene was; forward 5′‐GAAAGCTTGACCAAGCAAGCAC‐3′ and reverse 5′‐ACGGGACAGTACGTTAAGATGAACA‐3′, and GAPDH housekeeping gene was; forward 5'-TGTGTCCGTCGTGGATCTGA-3' and reverse 5'-TTGATGTTGAAGTCGCAGGAG -3'.

### Statistical analysis

Data were analyzed using the statistical package SPSS version 17. Data were presented as mean ± standard error. Analysis of variance (ANOVA) followed by post hoc tests was applied in normally distributed quantitative variables while non-parametric Kruskal–Wallis test and Mann–Whitney test were used for non-normally distributed quantitative variables [34]. *P* values less than 0.05 were considered statistically significant***.***

## Results

### Cell culture findings

**Fig. 1:**
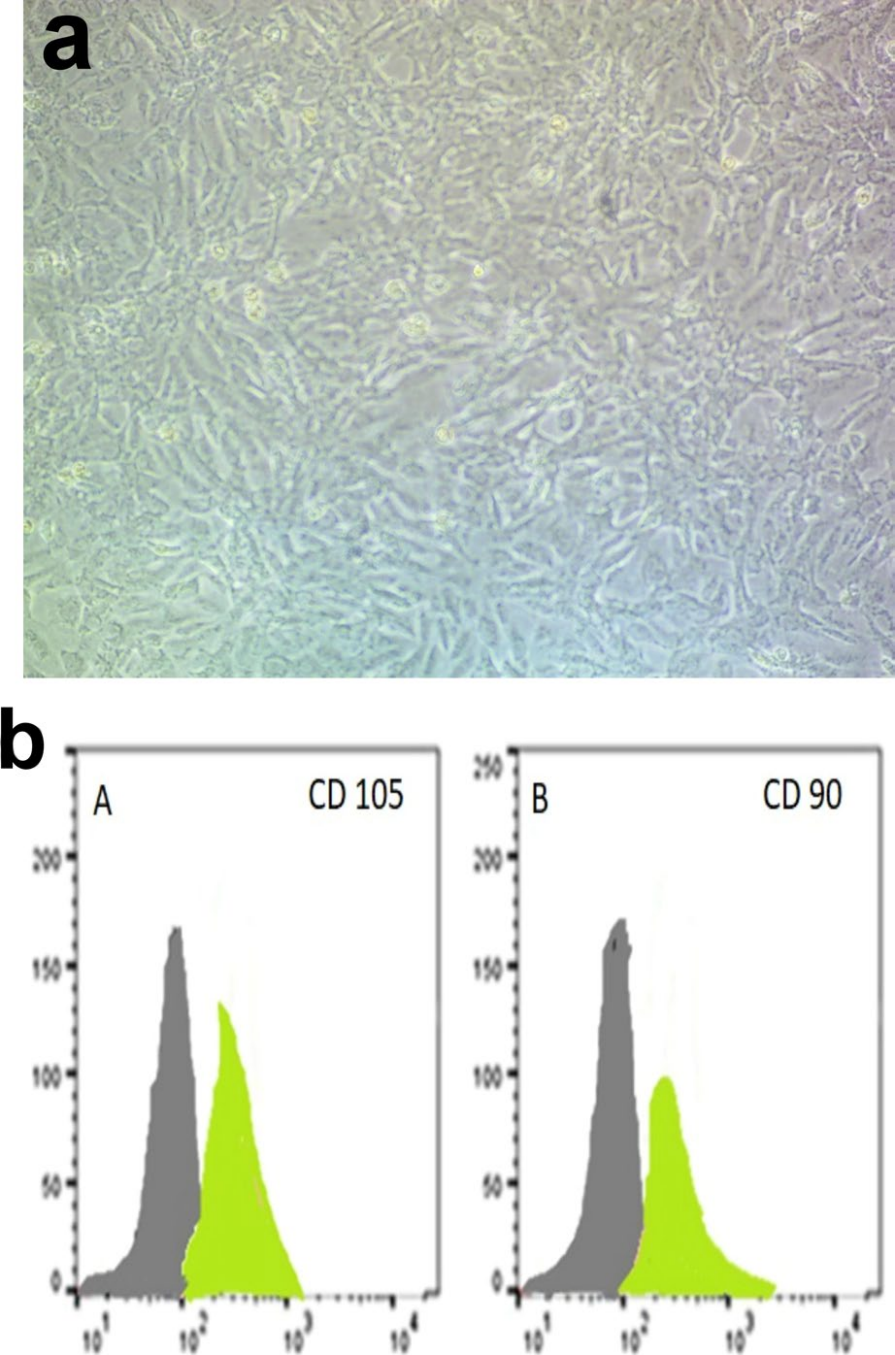
**a** Spindle-shaped camel Wharton’s jelly MSCs at two weeks of culture. **b** MSCS of camel characterization by FACS. **b** A: CD105 (97%) & B: CD90 (96.5%) respectively

Camel Wharton’s Jelly-derived MSCs were isolated, propagated in culture, and identified by their morphological fibroblast spindle-shaped cells (Fig. 1a). Analysis of MSCs based on cell surface marker expression for phenotypic identification revealed positive expression of CD105 (97%) & CD 90 (96.5%) in Camel MSCS characterization by Fluorescence-activated cell sorting (FACS) (Fig. 1b).

### Clinical results

#### Serum urea

In G1, the serum urea level increased gradually till the end of the experimental period reaching (71 ± 3.78 mg/dL). In G2, there was a marked increase in serum urea level till the 3rd week (55 ± 2.88 mg/dL) before CMSCs injection. The serum urea level begins to decrease from the 4th week till the end of the experiment reaching (26.6 ± 1.76* mg/dL) and recording a significant decrease compared to the values in G1. (Table 1).

**Table 1:** Serum urea concentration in different groups

Urea	zero time	1st week	2nd week	3rd week 1st dose of CMSCs	4th week	5th week 2nd dose of CMSCs	6th week	8th week	10th week	12th week
G1 (CKD)	13.33 ± 1.2	34 ± 2.08	43.66 ± 2.4	49.33 ± 2.9	60.33 ± 2.6	60 ± 2.88	64 ± 3.05	65.33 ± 2.9	67.66 ± 3.17	71 ± 3.78
G2 (camel WJ-MSCs)	12.33 ± 1.45	36.33 ± 1.76	43 ± 1.52	55 ± 2.88	38 ± 1.52*	37.33 ± 1.76*	34.66 ± 0.88*	33.33 ± 1.6*	27.66 ± 1.45*	26.6 ± 1.76*

Data are presented as mean value ± SE. * indicates significant difference at *P* value < 0.05

#### Serum creatinine

The serum creatinine level was significantly decreased at the 4th week just after the 1st stem cells injection. The value decreased from 3.22 to 2.59 and consequently reached 1.86 at the end of the experiment. (Table 2).

**Table 2:** Serum creatinine concentration in different groups

Creatinine	zero time	1st week	2nd week	3rd week 1st dose of CMSCs	4th week	5th week 2nd dose of CMSCs	6th week	8th week	10th week	12th week
G1 (CKD)	0.51 ± 0.03	0.74 ± 0.06	2.92 ± 0.09	3.14 ± 0.08	3.17 ± 0.13	3.23 ± 0.09	3.2 ± 0.11	3.23 ± 0.08	3.28 ± 0.1	3.32 ± 0.07
G2 (camel WJMSCs)	0.56 ± 0.05	0.85 ± 0.04	2.81 ± 0.04	3.22 ± 0.08	2.59 ± 0.1*	2.45 ± 0.1*	2.34 ± 0.08*	2.09 ± 0.13*	1.96 ± 0.07*	1.86 ± 0.08*

Data are presented as mean value ± SE. * indicates significant difference at *P* value < 0.05

#### KIM-1, NGAL, and EGFR genes expression

**Fig. 2:**
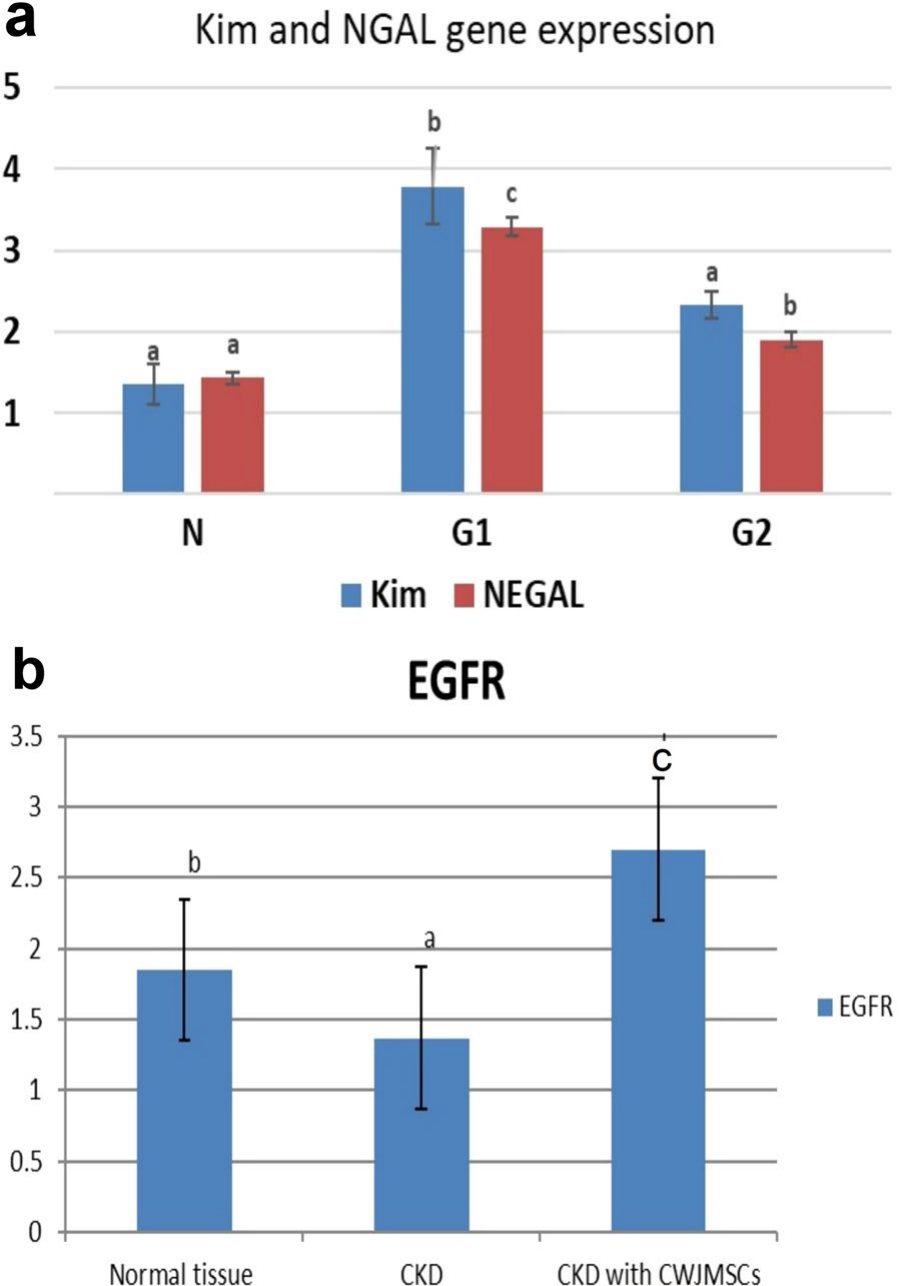
**a** KIM-1gene & NGAL gene expression in all experimental groups. **b** EGFR gene expression in all experimental groups. Columns bearing different lowercase letters are considered significant at *P* value < 0.05

At the end of the experimental period, NGAL gene expression in G2 was significantly decreased compared to G1 but it still showed a significant increase than normal. Kim-1 gene expression in G2 was significantly decreased compared to its value in G1, while no significant difference was recorded with normal tissue (Fig. 2a). On contrary, EGFR gene expression revealed a significant increase in G2 than G1 and normal tissue. (Fig. 2b)**.**

#### Histopathological findings

**Fig. 3:**
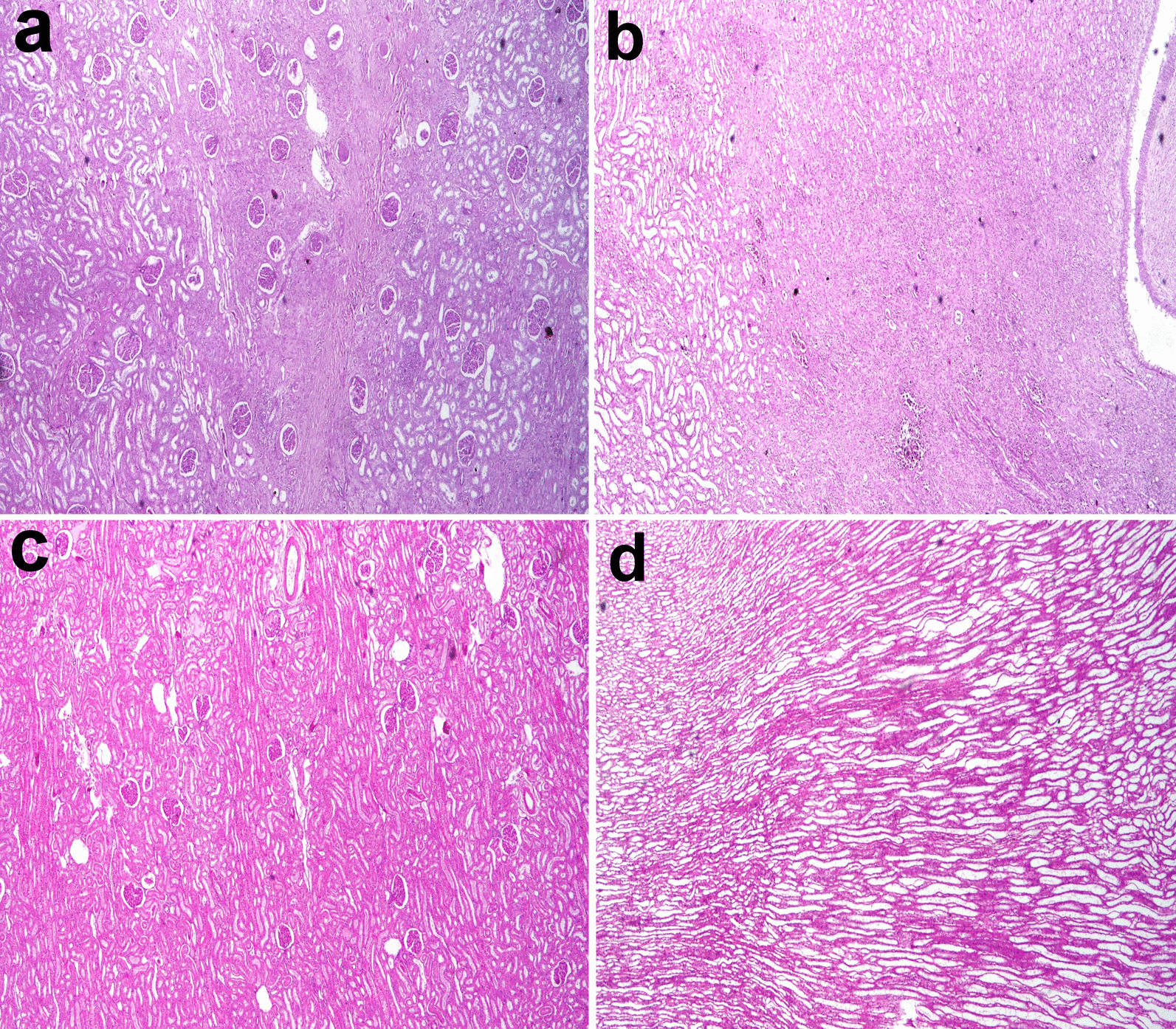
Kidney of a dog showing severe diffuse interstitial fibrosis in the (**a**) cortex and (**b**) medulla in G1. **c** mild histological changes in the cortex and **d** focal fibrosis in the medulla in G2 (H and E stain X40)

**Fig. 4:**
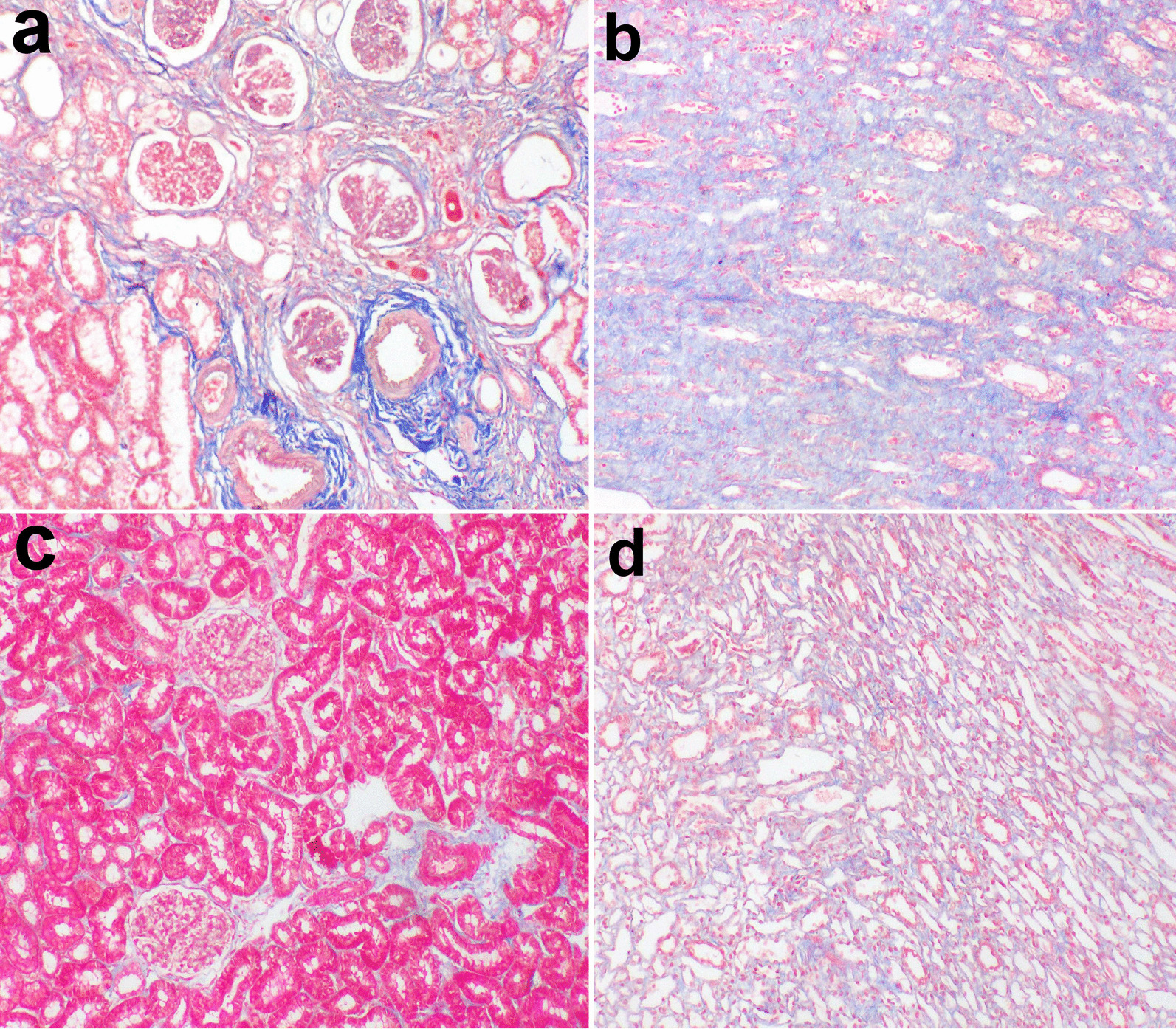
cortex and medulla of CKD model stained by MTC (**a**,**b**) and of treated cases with CWJMSCs (**c**, **d**)

**Fig. 5:**
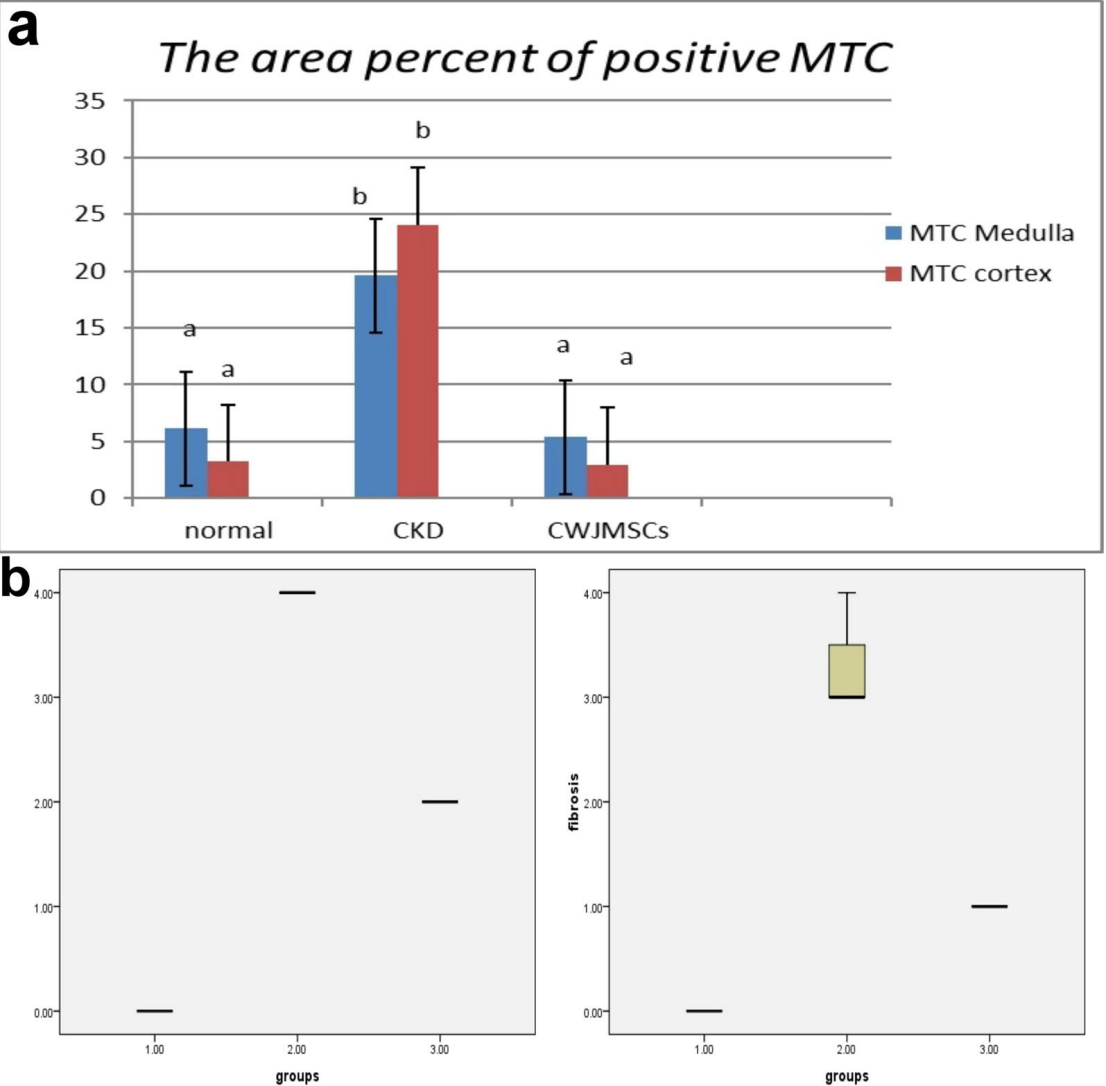
**a** the area percent of positive MTC in tissue sections of all experimental groups. Columns bearing different lowercase letters are considered significant at *P* value < 0.05. **b** Boxplot of glomerular lesion scores and fibrosis in different groups (1 = Normal group, 2 = G1, 3 = G2. The boxes are the interquartile range (IQR). The medians are the thick middle lines. The maximum and minimum values are represented by the thin horizontal lines at the top and bottom

The histological section of the kidney from the treated CKD model with CWJMSCs (G2) showed great improvement of glomerular lesions and tubular injury recorded in the CKD model (G1). The cortical and medullary fibrosis in G1 (Fig. 3a, b) became less extended in G2 (Fig. 3c, d) and this is confirmed by MTC stain (Fig. 4a–d). The area percent of fibrosis as demonstrated by MTC in the renal cortex and medulla of G1 was significantly higher than that in the normal and G2 (Fig. 5a).

**Fig. 6:**
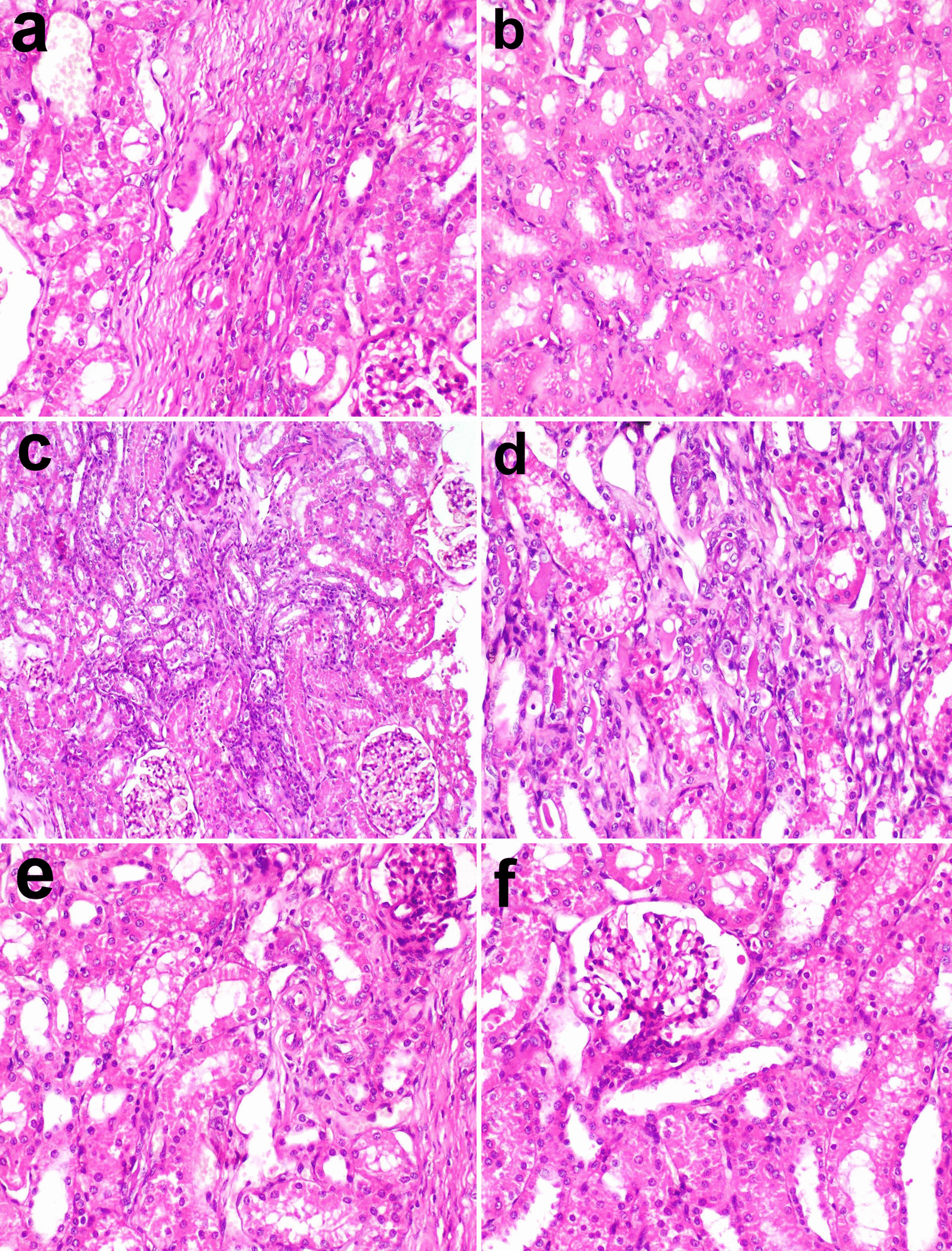
photomicrographs of kidney treated with CMSC. **a** Sheets of stem cells embedded in a strip of connective tissue at the injection site, **b** the stem cells scattered in the interstitial tissue between renal tubules, **c** focal area of regenerated tubules showing basophilia and nuclear crowding. Note the regenerated glomerulus (arrow), **d** focal area of remodeling showing newly formed blood vessels, scattered stem cells, regenerated tubules, and other degenerated ones. Note the denuded renal tubule and invaded with stem cells (arrow), **e** note the newly formed blood vessels in the cortex (arrow), **f** hyperplasia of juxtaglomerular cells (arrow)

The number of glomeruli that showed lesions were less than that observed in G1. The thickening of the glomerular basement membrane, mesangial hyperplasia, and bowman’s space exudation was less observed in the glomeruli as demonstrated by the lesion score of glomerular lesions. The lesion score of cortical and medullary glomerular lesions and fibrosis were significantly lower than the CKD group but was still significantly higher than the normal group (Fig. 5b). The stem cells appeared as a sheet of cells with different shapes, hyperchromatic nuclei embedded in a strip of connective tissue at the site of injection (Fig. 6a). They have attempted to arrange in a tubular structure, then get scattered in the interstitial tissue of the cortex and medulla (Fig. 6b). There were foci of regenerated tubules that showed nuclear crowding and sometimes binucleation (Fig. 6c). The regenerated cells appeared with basophilic cytoplasm, enlarged vesicular nuclei. Other foci showed remodeling with newly formed blood vessels, scattered stem cells in a basophilic matrix, regenerated and degenerated tubules (Fig. 6d). Remarkable newly formed blood vessels were usually scattered in the cortex (Fig. 6e) and prominent hyperplasia of juxtaglomerular cells was also recorded (Fig. 6f).

### PAS stain result

**Fig. 7:**
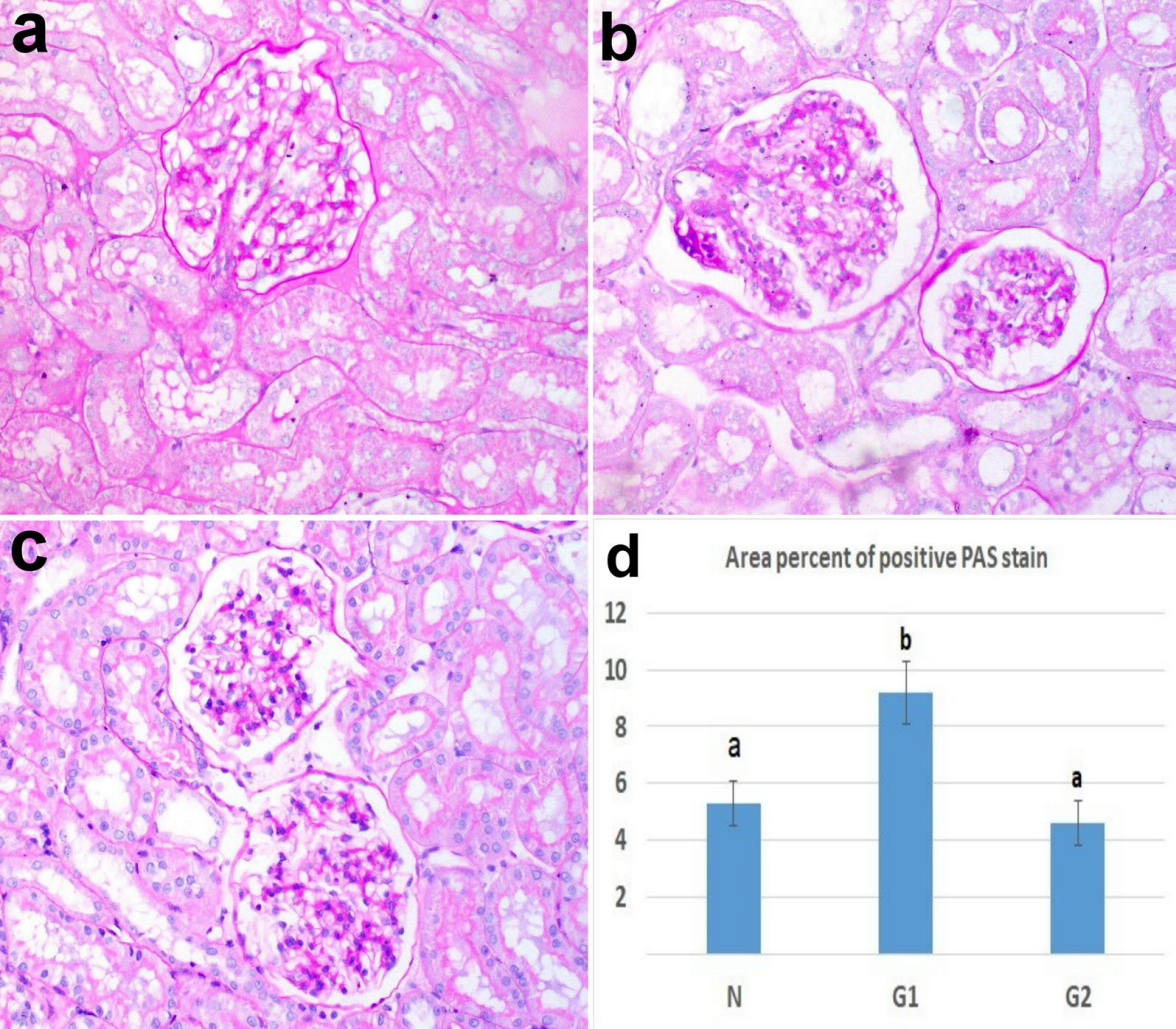
kidney tissue stained by PAS **a** control **b** CKD model **c** treated with CWJMSCs. Note the distinct brush border in (**c**). **d** the area percent of positive PAS in tissue sections of all experimental groups. Columns bearing different lowercase letters are considered significant at *P* value < 0.05

The stained sections of the kidneys by PAS showed the improvement and the integrity of kidney structure in G2 compared to the other groups, as the brush border of convoluted tubules was well distinct (Fig. 7a, b, c). The area percent of PAS stain in the CKD group was higher than the control group but it didn’t record significance. On the other hand, the area percent of PAS stain was significantly decreased in G2 compared to G1 (Fig. 7d).

### Immunohistochemistry of VEGF

**Fig. 8:**
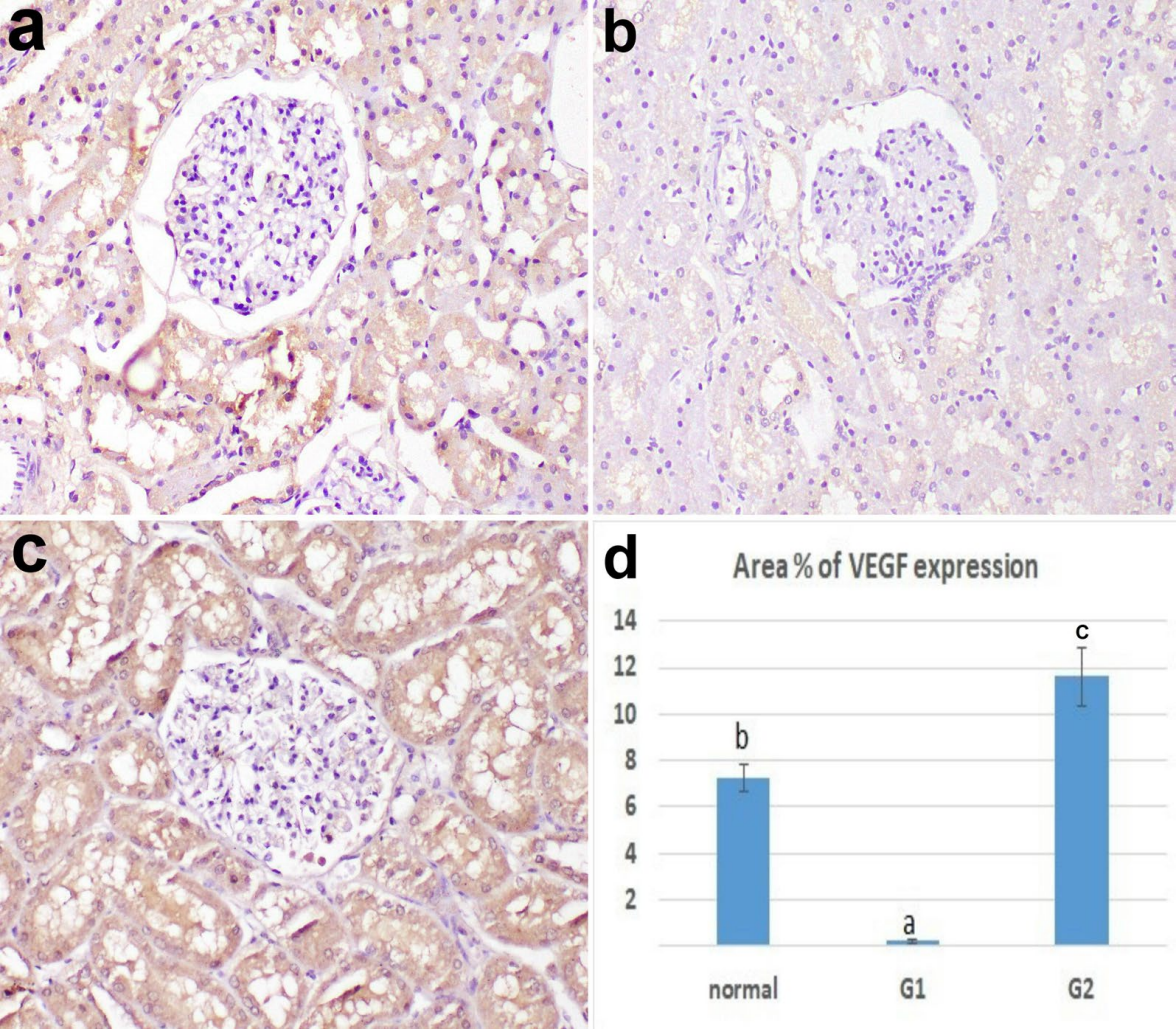
the expression of VEGF in the kidney tissue, **a** control, **b** CK model, **c** treated with CWJMSCs. **d** The area percent of VEGF expression in immunohistochemically stained sections in all experimental groups. Columns bearing different lowercase letters are considered significant at *P* value < 0.05

VEGF expression was mainly observed in the renal tubular epithelium. The area percent of its expression was significantly low in G1 compared to the control group. On the other hand, the area percent of VEGF expression in G2 was significantly high compared to control and G1 (Fig. 8).

## Discussion

The continuously asked question is; Can stem cells, especially the mesenchymal type, regenerate kidney tissue? The answer originates from the fact that the nephrons have a mesenchymal origin, so, MSCs can repair the injury in renal tissue and differentiate into collecting ducts and nephrons [35]. In the current study, after 12 weeks from CKD induction, the serum urea and creatinine levels were significantly high in the CKD model. It can be classified as stage 3 of CKD according to the staging system developed by IRIS [36]. Our clinical results agree with Ismail et al. [16] who recorded a significant increase in serum urea and creatinine levels after subtotal nephrectomy in dogs.

The dogs treated with CWJ MSCs showed improved kidney function tests after one week of stem cells injection and by the end of the experiment, they got classified as stage 2 of CKD according to the staging system developed by IRIS [36]. A similar finding has been reported by Villanueva et al., [37] who demonstrated that the intravenous infusion of adipose tissue mesenchymal stem cells (AD-MSC) has a role in kidney repair and improvement of renal function in rats with CKD. Furthermore, Semedo et al. [38] reported improvement in renal function of rats with CKD after bone marrow mesenchymal stem cells injection (BM-MSCs). On contrary, injection of allogeneic AD-MSC in CKD cats was not associated with adverse effects but didn’t improve renal function [39]. On the same occasion, the use of allogeneic mesenchymal stem cells (AMSCs) from the amniotic membrane in CKD cats improves renal function and disease progression [40]. The different results obtained in the studies of CKD cats may be attributed to the different types of MSCs used. A great similarity to the present study was obtained when I/V injection of human umbilical cord mesenchymal stem cells in a patient with chronic renal failure for 2 years was performed. It decreased her creatinine level from 11 mg/dL to 2 mg/dL after 8 months from the 1st stem cells injection [41]. In the present study, the improvement of kidney lesions is based on the new vascularization, tubular and glomerular regeneration, and decreasing fibrosis. VEGF expression was increased in the treated group with CWJMSCs as blood vessels remodeling occurred a matter which agreed with Gu et al. [42] who explained the ability of MSCs to differentiate into smooth muscle cells and endothelial cells, and secretion of various trophic factors including VEGF, enabling them to contribute to vascular regeneration through paracrine effects. A previous study showed that VEGF levels were substantially higher in BM-MSC treated rats with CKD induced by 5/6 nephrectomy after one month from MSC injection [43]. CWJMSCs invaded the interstitial tissue with an increased percent of regenerated renal tubules and retained the normal appearance of renal corpuscles. The renal protective effect of MSCs could be due to their capacity to engraft to the damaged kidney, integrate within renal tubules, and differentiate to renal tubular cells with reducing inflammation and fibrosis [44, 45]. Moreover, glomerulogenesis could occur with the stability of renal function [46].

The improvement of renal lesions in the treated group was accompanied by a decrease in the expression of KIM-1 and NGAL genes compared to the CKD group indicating renal tissue repair as these genes expression increases with increased inflammation, renal fibrosis, and chronically damaged tubular cells [47]. The renal tissue repair in this study was also confirmed by the result of EGFR gene expression which was significantly higher in the treated group than the CKD group. Our results agree with Villanueva et al. [37] whose study reported the ability of MSC to promote kidney repair by inducing the expression of repairing proteins that activate genes involved in the regeneration processes. Tang et al. and Isaka [1, 48] explained that EGF activates cells via the EGF receptor (EGFR), which is important in various biological processes including renal tubular cell regeneration, proliferation, and differentiation, in addition to accelerating the recovery of renal function after injury.

The most important study limitation was the inability to collect urine from dogs for albumin analysis. This was mainly due to the difficulty of catheterization in dogs for a long time and the vulnerability to infection.

**In Conclusion**: the transplantation of CWJ-MSCS in the CKD model induced in dogs can improve kidney function tests, have the power to regenerate damaged renal tubules, maintain the integrity of renal corpuscles, and decrease fibrosis.

## Data Availability

The datasets used and/or analyzed during the current study are available from the corresponding author on reasonable request.

## Abbreviations

CWJ-MSCS: Camel Whorton jelly-mesenchymal stem cells
CKD: Chronic kidney disease
RT-PCR: Realtime-polymerase chain reaction
NGAL: Neutrophil gelatinase-associated lipocalin (NGAL)
KIM-1: Kidney injury molecule
EGFR: Epidermal growth factor receptor
VEGF: Vascular endothelial growth factor
AD-MSC: Adipose tissue mesenchymal stem cells
BM-MSCs: Bone marrow mesenchymal stem cells injection.
AMSCs: Allogeneic mesenchymal stem cells
